# Burnout and Hypocortisolism – A Matter of Severity? A Study on ACTH and Cortisol Responses to Acute Psychosocial Stress

**DOI:** 10.3389/fpsyt.2015.00008

**Published:** 2015-02-02

**Authors:** Anna-Karin Lennartsson, Anna Sjörs, Peter Währborg, Thomas Ljung, Ingibjörg H. Jonsdottir

**Affiliations:** ^1^The Institute of Stress Medicine, Gothenburg, Sweden; ^2^Sahlgrenska Academy, University of Gothenburg, Gothenburg, Sweden; ^3^Department of Health Sciences, Mid Sweden University, Östersund, Sweden; ^4^The Department of Neuroscience and Physiology, Sahlgrenska Academy, University of Gothenburg, Gothenburg, Sweden

**Keywords:** chronic stress, burnout, Trier Social Stress Test, acute stress response, adrenocorticotropic hormone, cortisol, hypocortisolism

## Abstract

**Background:** Common consequences of long-term psychosocial stress are fatigue and burnout. It has been suggested that burnout could be associated with hypocortisolism, thus, inability to produce sufficient amounts of cortisol. This study aimed to investigate whether patients with clinical burnout exhibit aberrant ACTH and cortisol responses under acute psychosocial stress compared with healthy individuals.

**Methods:** Nineteen patients (9 men and 10 women) and 37 healthy subjects (20 men and 17 women), underwent the Trier Social Stress Test. Blood samples and saliva samples were collected before, after, and during the stress test for measurements of plasma ACTH, serum cortisol, and salivary cortisol. Several statistical analyses were conducted to compare the responses between patients and controls. In addition, in order to investigate the possibility that burnout patients with more severe symptoms would respond differently, sub-groups of patients reporting higher and lower burnout scores were compared.

**Results:** In both patients and healthy controls, we observed elevated levels of ACTH and cortisol after exposure to the stressor. There were no differences in responses of ACTH, serum cortisol, or salivary cortisol between patients and controls. Patients reporting higher burnout scores had lower salivary cortisol responses than controls, indicating that patients with more severe burnout symptoms may be suffering from hypocortisolism. In addition, patients with more severe burnout symptoms tended to have smaller ACTH responses than the other patients. However, there was no corresponding difference in serum cortisol.

**Conclusion:** This study indicates that hypocortisolism is not present in a clinical burnout patient group as a whole but may be present in the patients with more severe burnout symptoms.

## Introduction

Work-related stress is becoming one of the major challenging health issues among the European workforce (1). Common consequences of long-term stress are fatigue and burnout. Burnout can be defined as a negative affective state consisting of emotional exhaustion, cognitive weariness, and physical fatigue, which is caused by chronic psychosocial stress (2). Besides the mental health burden and consequences for quality of life, there is growing evidence that burnout can negatively influence physical health (2–4). Burnout leads to reduced work ability (5, 6) and is often associated with long-term sick leave (7, 8).

It has been suggested that burnout could be associated with hypocortisolism, thus, inability to produce sufficient amounts of cortisol (9). During stress exposure, the hypothalamic–pituitary–adrenal (HPA) axis is activated and the adrenal cortex produces high levels of cortisol. Cortisol serves as an energy mobilization hormone and elevated cortisol levels give us abilities to fight and overcome what makes us stressed. It is suggested that this hyperactivity of the HPA axis that occurs during stress can eventually turn into hypoactivity after long-term exposure to stressful circumstances without sufficient recovery (9). The inability to produce sufficient amount of cortisol is accordingly hypothesized to partly explain the fatigue and exhaustion symptoms of these individuals.

Studies on basal cortisol secretion, such as the cortisol awakening response or diurnal cortisol profiles, have so far not provided consistent evidence of the existence of hypocortisolism in burnout (10–13). However, differences between patients and controls are more likely to appear when the HPA axis is challenged (14), such as when the individual is exposed to an acute stress situation. The HPA axis response to a laboratory stress test (combined arithmetic and speech task) was investigated by De Vente et al. (15). They found no deviations in HPA axis reactivity and recovery during and after acute stress. However, the stress test did not induce a significant task-related cortisol response, which limited the possibility to draw any conclusions about hypocortisolism. The present study aims to investigate whether patients with clinical burnout exhibit aberrant cortisol and ACTH responses to acute psychosocial stress compared with healthy individuals using the well validated Trier Social Stress Test (TSST). In order to investigate the possibility that burnout patients with more severe symptoms would respond differently compared with other patients, the study also aimed to study sub-groups of burnout reporting higher and lower burnout scores. Measuring the ACTH response offers the possibility to broaden the knowledge on different levels of the HPA axis including the adrenal ACTH sensitivity. Blood pressure and heart rate changes in response to the stressor were also investigated to ensure that patients and controls exhibited a task-related stress response.

## Materials and Methods

### Participants

Nineteen individuals clinically diagnosed with burnout (9 men and 10 women) and 37 healthy subjects (20 men and 17 women), aged 31–50 years (mean age 39 years, SD 5.3 years) were included in the study. The patients were recruited from a specialist clinic, which exclusively treats patients with stress-related mental disorders, in the region of Västra Götaland, Sweden. The patients were originally referred to the stress clinic from primary health-care centers or occupational health service centers. They were ambulatory at the time of the study, and none had received in-patient care for their illness. All the patients fulfilled the diagnostic criteria for stress-related exhaustion disorder as previously described by Jonsdottir and co-workers (16) and had a maximal duration of sick leave of 6 months. Stress-related exhaustion disorder is a criteria-based diagnosis that has been used in Sweden since 2005 to define patients seeking health care for clinical burnout. The controls were recruited from a cohort study, surveying psychosocial work environment and health, and through advertising in a local daily newspaper. Only individuals reporting “no stress at all” or “only a little stress” on a single perceived stress item (17) were included as controls in order to avoid inclusion of individuals suffering from chronic stress problems. To be included in the study, subjects had to be between 30 and 50 years of age. For both patients and controls, exclusion criteria were having a body mass index (BMI) <18.5 or over 30 kg/m^2^, high blood pressure, infection, vitamin B-deficiency (high homocysteine), known systemic disease such as diabetes or thyroid disease or known psychiatric disease. As the menstrual cycle and the use of estrogens are known to affect the physiological response to acute stress (18, 19), women taking estrogens and postmenopausal women were excluded. Subjects who were taking psychoactive medications or any medications that might affect the HPA axis function, such as, for example, antidepressants, were excluded. Other exclusion criteria were being pregnant or nursing. The study was approved by the Regional Ethical Review Board in Gothenburg, Sweden, and was conducted according to the Helsinki Declaration. All participants gave written informed consent before entering the study.

### Study procedure

The participants underwent the TSST, a well-known standardized laboratory stress test. The TSST was set up according to the original design of Kirschbaum et al. (20). The stress task in TSST consists of a simulated job interview and a mental arithmetic task, both in front of a committee (two men and one woman), a video camera, and a microphone. Subjects were instructed to abstain from hard physical exercise 24 h before the stress test. Subjects were also instructed to avoid beverages containing caffeine at least 2 h before the stress test and to eat a standardized lunch. Smoking and using snuff were accepted but not on the test day. For female subjects, the stress tests were conducted between day 5 and 10 of the menstrual cycle (self-reported follicular phase). The stress tests were performed at the Institute of Stress Medicine of Region Västra Götaland in Gothenburg. The total test time for each subject was 2 h, including preparations and measurements after completing the test, and the test procedure was conducted between 13:00 and 17:00 hours (to avoid circadian rhythm effects). On arrival, an intravenous catheter was inserted in the subject’s forearm (−30 time point). The first blood sample was drawn at the −10 time point. The next blood sample was drawn directly before the TSST started (0 time point). Between these two measurements, the participants rested (approximately 7 min). At the start of the TSST, the participants were introduced to the tasks and asked to prepare for the simulated job interview (10 min). After this, the participants participated in the simulated job interview (5 min) and thereafter performed a mental arithmetic task (5 min). Directly after the end of the stress test (the +20 time point), a third blood sample was drawn. Thereafter, participants rested (recovery period of total 30 min), and 10 and 20 min into the recovery period, the fourth and fifth blood samples were drawn (+30 and +40 time points). A final blood sample was drawn at the end of the recovery period (+50 time point). Salivary samples were collected at eight time points (−10, 0, +10, +20, +30, +40, +50, and +60). Cardiovascular responses (heart rate, systolic blood pressure, and diastolic blood pressure) were electronically recorded (CardioPerfect Workstation, Welch Allyn) every fifth minute from 10 min before the TSST started until 30 min after the TSST ended (from the −10 time point to the +50 time point).

### Scoring of mental health

Several symptoms of mental health was measured to ensure that the controls were not suffering from mental health problems and to identify severity of symptoms among the patients. The Shirom-Melamed Burnout Questionnaire (SMBQ) (2) was used to measure burnout. SMBQ contains 22 items (graded 1–7) measuring the different aspects of burnout; emotional and physical exhaustion, tension, listlessness, and cognitive weariness. A mean burnout index was calculated for each participant. The index can range from 1 to 7. The SMBQ correlates strongly with the Maslach Burnout Inventory (21), the most widely used instrument for measurement of burnout. Clinical burnout in this study is set by using the diagnostic criteria of ED. However, as the ED criteria does not include grading of severity, scores from the SMBQ were used to define high and low severity of burnout among the patients, by using median split of the total scores. The hospital anxiety and depression (HAD) scale was used to assess self-reported depression and anxiety in both patients and controls. It was originally developed for non-psychiatric clinics to measure symptoms of depression (HAD-D) and anxiety (HAD-A) (22, 23), and the scale has been found to perform well in assessing anxiety disorders and depression in different patient groups as well as in the general population. Scores <7 indicate non-case for depression or anxiety, 7–10 indicate possible case for depression or anxiety, and scores above 10 on each respective subscale indicate probable case for depression or anxiety.

### Hormone assays

A total of 122 ml blood was collected from the participant during the TSST. Blood samples from six time points (−10, 0, +20, +30, +40, and +50) were collected (7 ml at each time point) for measurement of plasma ACTH and serum cortisol. The samples were collected in two different tubes; one pre-chilled containing EDTA and one free from EDTA. After the tubes had been centrifuged, plasma and serum were stored at −80°C until assayed. Salivary samples were collected at eight time points (−10, 0, +10, +20, +30, +40, +50, and +60) using Salivette tubes (Sarstedt, Nümbrecht, Germany). The saliva samples were then stored at −20°C until they were assayed. Plasma concentrations of ACTH were measured by immunoradiometric assay (limit of detection, 0.4 pmol/L) (CIS bio International, Gifsur-Yvette Cedex, France). Serum concentrations of cortisol were measured by electrochemiluminescence immunoassay (limit of detection, 20 nmol/L). Saliva concentrations of cortisol were measured using competitive radioimmunoassay (limit of detection, 1 nmol/L) (Spectria Coated Tube Radioimmunoassay; Orion Diagnostica, Espoo, Finland). Interassay coefficients of variation were below 10% for ACTH, 9% for serum cortisol, and 14% for salivary cortisol.

### Data handling

Baseline values for ACTH and cortisol were calculated as means of the values determined at the −10 and the 0 time points. Two men and one woman had missing values for heart rate as well as systolic and diastolic blood pressure at the 0 time point. For these three individuals, the −10 time point values for heart rate, systolic blood pressure, and diastolic blood pressure were used as baseline values. Area under the curve with respect to increase (AUC_I_) was calculated for ACTH, serum cortisol, and salivary cortisol (24). The Kolmogorov–Smirnov test was used on each study variable to test whether the data were normally distributed. Variables that showed a non-normal distribution were converted by logarithmic transformation.

### Statistical analyses

To evaluate possible differences between patients and healthy controls at baseline, age (log), BMI, and baseline values for ACTH (log), serum cortisol (log), salivary cortisol (log), heart rate, systolic blood pressure (log), and diastolic blood pressure were analyzed by using the *t*-test. Scores on the burnout, anxiety, and depression scales were analyzed by using the Mann–Whitney *U*-test. To investigate the HPA axis and cardiovascular reactions, paired sample *t*-tests were performed using before and after stress test values (baseline value and identified peak value), separately in patients and controls. Log-transformed concentrations of ACTH, serum cortisol, salivary cortisol, and systolic blood pressure were used in the analysis. To evaluate differences between patients and controls in HPA axis activation, mixed between–within ANOVAs (time × group) were performed on ACTH, serum cortisol, and salivary cortisol. Log-transformed concentrations were used in the analyses. Mann–Whitney *U*-test was used to compare AUC_I_ values for ACTH, serum cortisol, and salivary cortisol between patients and controls. To analyze whether patients with a higher degree of burnout symptoms responded differently from the other patients, the patients were divided into two sub-groups based on the median split (SMBQ: 4.55). AUC_I_ values for ACTH, serum cortisol, and salivary cortisol were compared between (a) patients with higher and lower burnout scores, (b) controls and patients with higher burnout scores, and (c) controls and patients with lower burnout scores using Mann–Whitney *U*-test. A non-parametric test was used in the comparisons of AUC_I_ between the sub-groups since the AUC_I_ measures were not normally distributed and logarithmic transformations were not successful in order to make some of the AUC_I_ measures normally distributed. For all tests, the level of significance was set at *p* < 0.05. Analyses were conducted with IBM Statistics 20 (SPSS Inc., Chicago, IL, USA).

## Results

### Study participants

Background characteristics of the patients and healthy controls are reported in Table 1. None of the hormonal and cardiovascular baseline levels measured before the TSST differed significantly between patients and controls (data not shown).

**Table 1:** **Characteristics of the patients with clinical burnout and healthy controls**.

	Patients		Controls		*p* Value
	*N*	Mean (range)	*N*	Mean (range)	
Number of Men/Women		9/10		20/17	0.779
Age (years)	19	40.6 (31−50)	37	37.5 (31−49)	**0.019**
BMI (kg/m^2^)	19	24.2 (19.2−30)	37	23.4 (18.5−30.1)	0.322
Burnout score (SMBQ)	19	4.4 (2.0−6.2)	36	2.5 (1.2−5.1)	**<0.001**
Depression score (HAD-D)	18	6.6 (1−13)	36	2.0 (0−9)	**<0.001**
Anxiety score (HAD-A)	18	9.3 (3−15)	36	4.3 (0−14)	**<0.001**

*p Value in bold indicate significant difference (p < 0.05). BMI, body mass index; SMBQ, Shirom-Melamed Burnout Questionnaire; HAD, Hospital Anxiety and Depression Scale*.

### Physiological response to the acute psychosocial stressor

**Figure 1:**
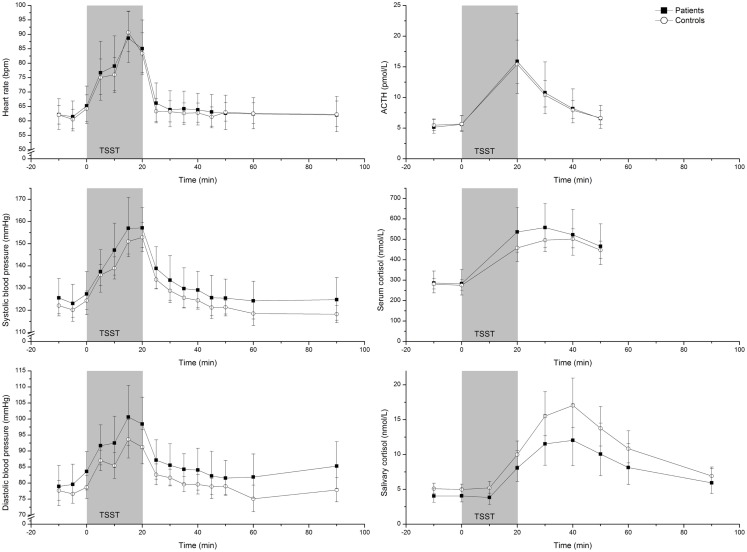
**Geometric mean (95% CI) heart rate, systolic blood pressure, diastolic blood pressure, ACTH, serum cortisol, and salivary cortisol concentrations in 19 patients and 37 healthy controls before, during, and after the Trier Social Stress Test**.

Baseline and peak values in the patients and controls are reported in Table 2. All parameters increased during the stress test both in the healthy controls and in the patients. Mean values of ACTH, cortisol, heart rate, systolic blood pressure, and diastolic blood pressure at each time point before, during, and after the stress test in the patients and controls separately are shown in Figure 1.

**Table 2:** **Physiological response to acute psychosocial stress in patients with clinical burnout and healthy controls**.

	Baseline	Peak	Paired samples *t*-test			
	Mean (range)	Mean (range)	*t*	*df*	*p* Value	Eta squared
**Patients**
P-ACTH (pmol/L)	5.98 (2.70−16.5)	21.6 (4.60−58.0)	−7.9	17	**<0.001**	0.79
S-cortisol (nmol/L)	307 (160−660)	618 (220−1200)	−9.3	14	**<0.001**	0.86
Sal-cortisol	4.52 (1.35−11.7)	16.5 (4.00−41.0)	−7.9	17	**<0.001**	0.79
Heart rate (bpm)	64 (44−88)	94 (67−144)	−8.8	17	**<0.001**	0.82
SBP (mmHg)	127 (100−159)	166 (140−205)	−9.3	15	**<0.001**	0.85
DBP (mmHg)	82 (62−101)	107 (86−133)	−10	17	**<0.001**	0.85
**Controls**
P-ACTH (pmol/L)	6.87 (1.85−23.9)	18.9 (3.20−49.0)	−11	38	**<0.001**	0.76
S-cortisol (nmol/L)	288 (185−570)	562 (290−880)	−17	35	**<0.001**	0.89
Sal-cortisol	5.57 (2.90−13.4)	21.6 (5.90−52.0)	−12	37	**<0.001**	0.80
Heart rate (bpm)	64 (47−87)	96 (67−133)	−10	37	**<0.001**	0.77
SBP (mmHg)	123 (106−144)	161 (129−213)	−17	37	**<0.001**	0.89
DBP (mmHg)	79 (62−96)	100 (68−116)	−13	37	**<0.001**	0.82

*p Value in bold indicate significant difference (p < 0.05). *p* Values are adjusted for multiple comparisons (Bonferroni). P-ACTH, adrenocorticotropic hormone measured in plasma; S-cortisol, cortisol measured in serum; Sal-cortisol, cortisol measured in saliva; SBP, systolic blood pressure; DBP, diastolic blood pressure. Baseline levels of ACTH, cortisol, heart rate, SBP, and DBP are the average of the level at the −10 and the 0 time point. The peak level are the highest levels reached between the +20 and +50 time points, for ACTH and cortisol, and between the +5 and +20 time point for heart rate and blood pressure*.

### Differences in HPA axis response between patients and controls

Mixed between–within ANOVAs (time × group) were performed for ACTH, serum cortisol, and salivary cortisol separately. There was a substantial main effect for time in the analysis of ACTH [*F*(1, 57) = 119, *p* < 0.001, partial eta squared = 0.898], serum cortisol [*F*(1,50) = 69, *p* < 0.001, partial eta squared = 0.854], and salivary cortisol [*F*(1,53) = 29, *p* < 0.001, partial eta squared = 0.810]. Thus, levels of ACTH, serum cortisol, and salivary cortisol changed significantly over time. There was no interaction effect in the analysis of ACTH (*p* = 0.872), serum cortisol (*p* = 0.837), or salivary cortisol (*p* = 0.958), thus the response patterns were similar in patients and controls. There was no effect of group in the analysis of ACTH (*p* = 0.591), serum cortisol (*p* = 0.388), or salivary cortisol (*p* = 0.121), thus levels of ACTH, serum cortisol, and salivary cortisol during the experiment were not significantly different between patients and controls. Furthermore, there were no differences between patients and controls in area under the curve with respect to increase (AUC_I_) in ACTH (AUC_I_ = 365 and 263, respectively; *Z* = −0.332 *p* = 0.740), serum cortisol (AUC_I_ = 10509 and 8553, respectively; *Z* = −1.140, *p* = 0.254), or salivary cortisol (AUC_I_ = 476 and 633, respectively; *Z* = −1.127, *p* = 0.260).

### Difference in HPA axis response between patients with higher and lower symptomatology

**Figure 2:**
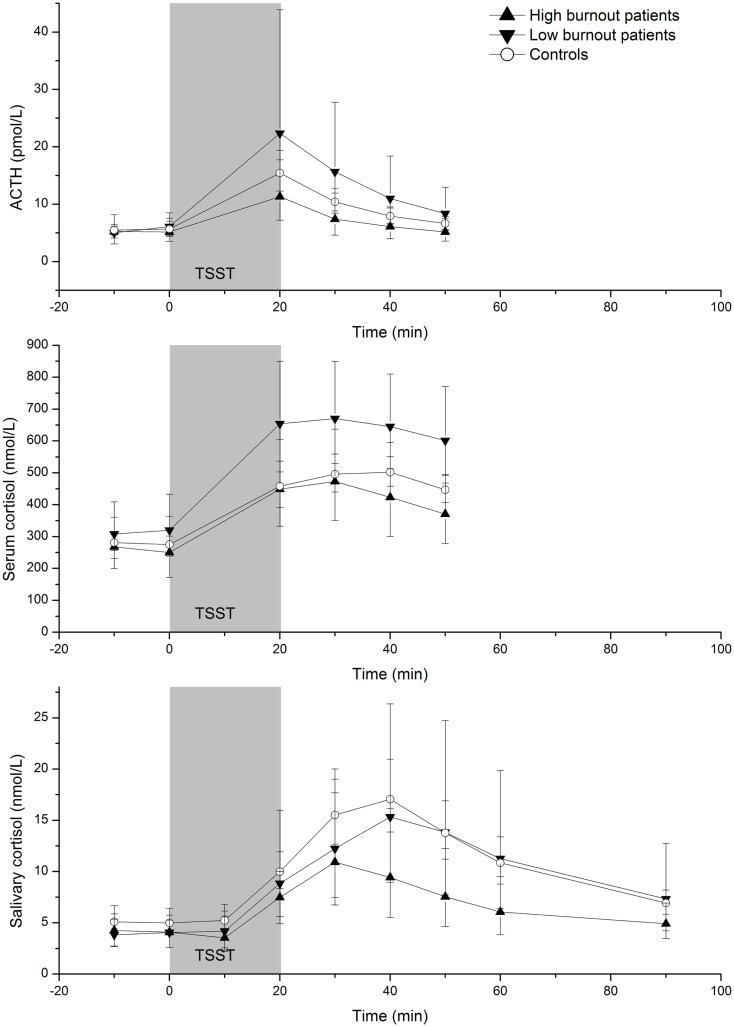
**Geometric mean (95% CI) ACTH, serum cortisol, and salivary cortisol concentrations before, during, and after the Trier Social Stress Test in patients with higher (*n* = 10) and lower (*n* = 9) burnout scores and controls (*n* = 37)**.

To further explore the patient group, the patients were divided into two sub-groups based on the scores on the burnout questionnaire (median split). Figure 2 illustrates the levels of ACTH, serum cortisol, and salivary cortisol at each time point for the controls and the patients with higher and lower burnout scores. The patients with higher burnout scores showed a lower response to the acute stressor (AUC_I_) of serum cortisol (AUC_I_ = 7288 and 13731, respectively; *Z* = −2.21, *p* = 0.027) than the patients with lower burnout scores. Although the corresponding differences were in the same direction for ACTH (AUC_I_ = 142 and 588, respectively; *Z* = −1.63, *p* = 0.070) and salivary cortisol (AUC_I_ = 229 and 754, respectively; *Z* = −1.72, *p* = 0.068), these differences failed to reach statistical significance. When comparing the two groups of patients with the control group, it was seen that the patients with higher burnout scores had a lower response (AUC_I_) of salivary cortisol compared to the controls (AUC_I_ = 229 and 633, respectively; *Z* = −2.20, *p* = 0.028) but there were no significant differences in ACTH response (AUC_I_ = 142 and 263, respectively; *Z* = −1.18, *p* = 0.239) or serum cortisol response between patients with higher burnout scores and controls (AUC_I_ = 7288 and 8553, respectively; *Z* = −1.07, *p* = 0.287). The patients with lower burnout scores had, compared to the controls, larger AUC_I_ for serum cortisol (AUC_I_ = 13731 and 8553, respectively; *Z* = −2.82, *p* = 0.005) but no statistical significant differences in ACTH AUC_I_ (AUC_I_ = 588 and 263, respectively; *Z* = −1.69, *p* = 0.091) or salivary cortisol AUC_I_ (AUC_I_ = 754 and 633, respectively; *Z* = −0.564, *p* = 0.573).

## Discussion

This study aimed to investigate whether patients with clinical burnout exhibited hypocortisolism since this is believed to constitute an underlying mechanism behind the fatigue symptoms in the burnout syndrome. This was studied through exposing the patient and healthy controls to an acute psychosocial stressor, since differences in cortisol production capacity are easier to detect when testing the reactivity instead of resting levels. Levels of cortisol and ACTH, as well as blood pressure and heart rate, increased markedly in both patients and controls in response to the acute psychosocial stressor. The different statistical analyses that were conducted to compare the ACTH and cortisol responses between patients and controls revealed no differences between the groups. In order to investigate the possibility that burnout patients with more severe symptoms would respond differently than the other patients, statistical analyses were also performed using the sub-groups with higher and lower burnout scores. Salivary cortisol responses were lower in the patients with high burnout scores than the controls, indicating that patients with more severe burnout symptoms may be suffering from hypocortisolism. However, there was no corresponding difference in serum cortisol. The correlation between concentrations of cortisol in serum and salivary are considered to be high (25) but during situations when serum concentrations are elevated (26) such as during an acute stress situation, the correlation is lower. This has been shown to be due to changed levels of corticosteroid-binding globulin levels during these situations (27, 28), which in turn affects the serum cortisol concentrations (29). Thus, when measuring cortisol during acute stress situations, changes in salivary cortisol levels might be a better indicator of cortisol secretion than changes in serum cortisol levels.

In addition to lower salivary cortisol responses in the high burnout patients, a trend to lower ACTH responses was also seen. This indicates that the lower salivary cortisol responses in the high burnout patients do not seem to depend on adrenal desensitization to ACTH. This needs to be confirmed in larger studies.

As mentioned in the Section “Introduction,” there are several previous studies, which have investigated basal cortisol levels in burnout. These studies show inconsistent results. Studies on the cortisol awakening response have mostly revealed no difference between patients and controls (11–13). Evening cortisol levels have been found to be either equal (12) or decreased (13) compared with controls. De Vente et al. (15) investigated the HPA axis reactivity in burnout patients in response to an acute stressor. They used a modified version of the TSST, which did not induce any significant elevation in cortisol levels. Hence, it is difficult to draw any conclusions about hypocortisolism in burnout from that study. Taken together, neither previous research on basal cortisol and diurnal cortisol nor the present study on cortisol reactivity provides any clear evidence for hypocortisolism in burnout. Instead, the research, including the present study, points toward a diversity of cortisol level and HPA axis activity function among sub-groups in this patient group.

It should be mentioned that difficulties to recruit patients limited the sample size and hence also the conclusion that can be drawn from this study. The main difficulty encountered during recruitment of patients was the use of antidepressants as one of the exclusion criteria. Antidepressants have been shown to affect the HPA axis reactivity (30) and unfortunately most of the patients who were referred to the stress clinic were already taking antidepressants. It is therefore important to note that this study should be considered as a pilot study. Another issue is possible sex differences. The patient group and the control group consist of equal proportions of men and women. The sub-group of patients with lower burnout scores, however, consists of a larger proportion of men than the high burnout score sub-group does. Since it may be a difference in responses between the sexes in general, this difference in proportions may affect the results for the sub-group analyses. Separate analyses of men and women (comparisons of AUC values) do, however, show the same pattern as when the analyses are performed with men and women together. The number of cases is, however, small and larger studies are needed for firm conclusions.

## Conclusion

This study indicates that hypocortisolism is not present in a clinical burnout patient group as a whole but may be present in the patients with more severe burnout symptoms. Since there are a small number of patients in the sub-groups analysis with higher and lower burnout symptoms, these results need to be confirmed by larger studies. If this is the case, some of the inconsistencies in the literature could be explained by the fact that different severities of burnout symptoms have been studied. Other mechanisms behind the stress-related fatigue in patients with burnout/exhaustion than hypocortisolism should also be explored.

## Conflict of Interest Statement

The authors declare that the research was conducted in the absence of any commercial or financial relationships that could be construed as a potential conflict of interest.
